# Real-World Data of First-Line Cemiplimab Monotherapy for Metastatic Non-Small Cell Lung Cancer (NSCLC) with PD-L1 Expression ≥ 50%: A National Spanish Multicentric Cohort (CEMI-SPA Study)

**DOI:** 10.3390/cancers17223643

**Published:** 2025-11-13

**Authors:** Silvia Masini, Monica Antoñanzas Basa, Antonio Calles, Ruth Alvarez Cabellos, Ibone De Elejoste Echebarria, Cristina Traseira Puchol, Mireia Martinez Kareaga, Luis Cabezon-Gutierrez, Maria Corina Escoin Perez, Yolanda Lage, Ester Garcia Lorenzo, Fatima Navarro, Maria Sereno, Sandra Falagán Martínez, Carme García-Benito, Laura Masfarre Pinto, Claudio Avila Andrade, Silvia Sequero, Joaquín Mosquera Martinez, Ana López-Martín, Aitor Azkárate Martínez, Maria Cruz Martín-Soberón, Clara Lucia-Gozalvez, Judit Rubio, Leopoldo Tallafigo, Alberto Garrido, Melina Peressini, Javier Torres-Jimenez, María Zurera, Helena Bote, Santiago Ponce, Luis Paz-Ares, Jon Zugazagoitia, Javier Baena

**Affiliations:** 1 Medical Oncology Department, Hospital 12 de Octubre, 28041 Madrid, Spain; silvia.masini@humanitas.it (S.M.); javier.torres@salud.madrid.org (J.T.-J.); maria.zurera@salud.madrid.org (M.Z.); helena.bote@salud.madrid.org (H.B.); santiago.ponce@oncosur.org (S.P.); lpazaresr@seom.org (L.P.-A.); j.zugazagoitia.imas12@h12o.es (J.Z.); 2 Medical Oncology Department, Humanitas University, 20072 Milan, Italy; 3 Medical Oncology Department, Hospital Clinico Universitario San Carlos, 28040 Madrid, Spain; monica.antonanzas@salud.madrid.org; 4 Medical Oncology Department, Hospital Gregorio Marañón, 28007 Madrid, Spain; antonio.calles@salud.madrid.org; 5 Medical Oncology Department, Hospital Universitario de Toledo, 45007 Toledo, Spain; ruthalvarez21@gmail.com; 6 Medical Oncology Department, Hospital Universitario Donostia, 20014 Donostia, Spain; ibone_e@hotmail.com; 7 Medical Oncology Department, Hospital Universitario del Henares, 28822 Madrid, Spain; cristina.traseira@salud.madrid.org; 8 Medical Oncology Department, Hospital Universitario Araba, 01009 Araba, Spain; mireia.martinezkareaga@osakidetza.eus; 9 Medical Oncology Department, Hospital Universitario de Torrejón, 28850 Madrid, Spain; pitucgp@hotmail.com; 10 Medical Oncology Department, Hospital Universitario de la Ribera, 46600 Alzira, Spain; cor_esc@hotmail.com; 11 Medical Oncology Department, Hospital Ramón y Cajal, 28034 Madrid, Spain; yo_lage@hotmail.com; 12 Medical Oncology Department, START Madrid-FJD, Fundación Jiménez Díaz University Hospital, 28040 Madrid, Spain; ester.garcia@startmadrid.com; 13 Medical Oncology Department, Hospital Universitario Principe de Asturias, 28805 Madrid, Spain; fatimanavarro_md@yahoo.es; 14 Medical Oncology Department, Hospital Universitario Infanta Sofía, 28702 Madrid, Spain; maria.sereno@salud.madrid.org (M.S.); sfalagan@gmail.com (S.F.M.); 15 Medical Oncology Department, Complexo Hospitalario Universitario de Ourense, 32005 Ourense, Spain; carme.cgb@gmail.com; 16 Medical Oncology Department, Hospital del Mar, 08003 Barcelona, Spain; lauramasfarre@vhio.net; 17 Medical Oncology Department, Hospital Lluis Alcanyis de Xativa, 46800 Valencia, Spain; avila_claand@gva.es; 18 Medical Oncology Department, Hospital Universitario San Cecilio, 18007 Granada, Spain; silsq90@gmail.com; 19 Medical Oncology Department, Complexo Hospitalario Universitario A Coruña, 15006 A Coruña, Spain; joaquin.mosquera.martinez@gmail.com; 20 Medical Oncology Department, Hospital Universitario Severo Ochoa, 28914 Leganés, Spain; ana5lomar@gmail.com; 21 Medical Oncology Department, Hospital Universitario Son Espases, 07120 Mallorca, Spain; aitor.azkarate@ssib.es; 22 Medical Oncology Department, Hospital Universitario Infanta Elena, 28342 Valdemoro, Spain; mmsoberon@salud.madrid.org; 23 Medical Oncology Department, Hospital Universitario Sant Joaun de Reus, 43204 Reus, Spain; claraluciagozalvez@gmail.com; 24 Medical Oncology Department, Hospital Universitario de Móstoles, 28935 Móstoles, Spain; judit.rubio@salud.madrid.org; 25 Medical Oncology Department, Hospital Universitario de Jerez, 11407 Jerez, Spain; fernando.t.moreno@gmail.com; 26 Medical Oncology Department, Hospital Universitario de Pontevedra, 36071 Pontevedra, Spain; garrido.fernandez.alberto@gmail.com; 27 Instituto de Investigación Hospital 12 de Octubre, 28041 Madrid, Spain; melinaperessini.imas12@h12o.es

**Keywords:** immunotherapy, NSCLC, PD-L1

## Abstract

**Background/Objectives:** Anti–PD-1/PD-L1 blockers have revolutionized the treatment landscape of non–small cell lung cancer (NSCLC) lacking oncogene-addicted alterations, particularly in tumors with high PD-L1 expression (tumor proportion score [TPS] ≥ 50%). Cemiplimab is approved as first-line monotherapy in this setting. However, real-world data remain scarce. This study aimed to evaluate the efficacy and safety of single-agent cemiplimab in a multicenter Spanish cohort and compare outcomes with a historical pembrolizumab cohort. **Methods:** Cemi-SPA is a retrospective multicenter study including 150 patients with advanced NSCLC and PD-L1 ≥ 50% treated with cemiplimab as first-line monotherapy across 21 Spanish centers. Clinical outcomes were analyzed and compared with a historical cohort of 144 patients treated with pembrolizumab. Propensity score matching (PSM) was performed to adjust for baseline differences. **Results:** Median progression-free survival (PFS) and overall survival (OS) were 8.1 and 12.6 months, respectively. ECOG performance status ≥ 2 was independently associated with worse outcomes, whereas the development of immune-related adverse events correlated with improved PFS and OS. After PSM, no significant differences were observed between cemiplimab and pembrolizumab in terms of efficacy. **Conclusions:** Cemiplimab demonstrated comparable real-world efficacy and safety to pembrolizumab in patients with advanced NSCLC and PD-L1 ≥ 50%. ECOG performance status emerged as the strongest prognostic factor, highlighting the importance of patient selection in routine clinical practice.

## Simple Summary

Immunotherapy has changed how we treat patients with advanced non-small cell lung cancer, especially those whose tumors show high levels of PD-L1, a marker that helps predict response to treatment. Cemiplimab is an immunotherapy drug approved for these patients based on results from clinical trials. However, information about how cemiplimab works in everyday clinical practice is lacking. In this study, we analyzed data from 150 patients treated at 21 hospitals across Spain to gain a better understanding of how cemiplimab performs outside the controlled environment of clinical trials. We also compared these results with a separate group of patients previously treated with another commonly used drug, pembrolizumab. Our findings show that cemiplimab is effective and safe in routine care and works similarly to pembrolizumab. Importantly, we confirmed that a patient’s overall health and physical condition strongly influence treatment outcomes, helping guide future treatment decisions.

## 1. Introduction

Immunotherapy has redefined the treatment landscape of cancer [1]. Among the different immune checkpoint inhibitors (ICIs), anti-PD-1/PD-L1 therapy has become a cornerstone in the management of several oncological diseases, including NSCLC. In this malignancy, these agents have shown robust efficacy across multiple treatment scenarios, particularly in the first-line treatment of advanced disease [2,3].

The selection of first-line systemic anticancer therapy for unresectable advanced NSCLC is guided by a multifaceted assessment of different tumor features, including histology, PD-L1 expression and conquering actionable molecular alterations, along with patient-related factors such as PS-ECOG and comorbidities, together with individual preferences [4].

For patients without EGFR, ALK, or ROS-1 molecular aberrations, standard-of-care regimens include chemotherapy combined with ICIs (anti-PD-1/PD-L1, with or without anti–CTLA-4 antibodies). Furthermore, the subset of patients whose tumors exhibit high PD-L1 expression, as defined by a tumor proportion score (TPS) ≥ 50% are more likely to derive significant benefit from these regimens. Consequently, the efficacy of single-agent anti-PD-1/PD-L1 therapies has been evaluated in this subgroup with positive results, and several agents, including pembrolizumab, atezolizumab, and cemiplimab, have received regulatory approval for this indication [5,6,7].

Cemiplimab is a fully human IgG4 monoclonal antibody targeting PD-1. In the phase III EMPOWER-Lung 1 trial, single-agent cemiplimab has shown superior efficacy and improved overall survival (OS) compared to chemotherapy in patients with advanced NSCLC and high PD-L1 expression. The recently published 5-year update confirmed the durability of clinical benefit, reporting a more than twofold higher objective response rate (46.5% vs. 20.6%), as well as significant improvements in progression-free survival (PFS; 8.1 vs. 5.3 months; HR 0.50) and OS (26.2 vs. 13.3 months; HR 0.585). A positive association between the level of PD-L1 expression and clinical benefit was also observed. The safety profile was consistent with other PD-1 or PD-L1 agents, with grade ≥ 3 immune-related adverse events (irAEs) occurring in 18.3% of patients. Treatment discontinuation due to toxicity occurred in 4.2%, and 2.8% of patients experienced treatment-related deaths [7].

Despite these encouraging results, real-world data (RWD) on cemiplimab remain scarce. Clinical trials are often conducted in selected populations within academic centers under controlled conditions, which may not accurately reflect everyday clinical practice. In contrast, real-world patients tend to be older, have more comorbidities, and present with poorer functional status or logistical barriers to care. Therefore, observational studies in real-life settings are essential to better understand the generalizability of trial results and to characterize treatment outcomes in more heterogeneous populations.

This study aims to evaluate real-world clinical outcomes in a multicenter Spanish cohort of patients with advanced NSCLC and high PD-L1 expression treated with single-agent cemiplimab in the first-line setting. We also performed an indirect comparison with a historical multicenter cohort of patients treated with pembrolizumab and conducted a pooled analysis to identify clinical factors associated with treatment efficacy.

## 2. Materials and Methods

### 2.1. Study Population and Data Source

**Figure 1:**
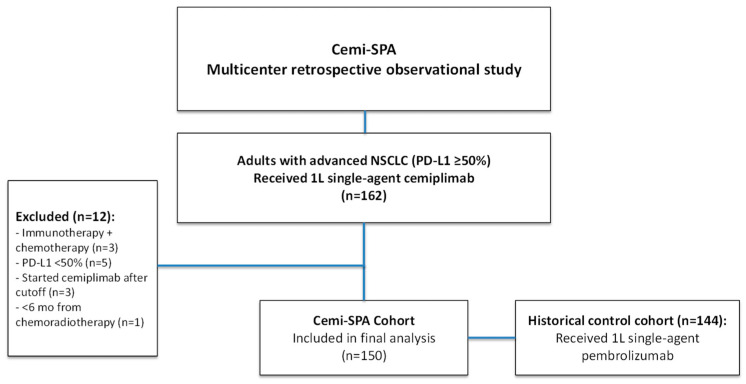
Consort Diagram. Flowchart detailing patient inclusion in the Cemi-SPA study. A total of 162 patients with advanced NSCLC and PD-L1 ≥ 50% were initially screened. Twelve patients were excluded due to prior chemo-immunotherapy (n = 3), PD-L1 expression < 50% (n = 5), cemiplimab initiation after the study cutoff date (n = 3), or initiation within 6 months of previous chemoradiotherapy (n = 1). A final cohort of 150 patients receiving first-line single-agent cemiplimab was included in the safety and efficacy analysis. A parallel historical control cohort of 144 patients treated with first-line single-agent pembrolizumab was also analyzed for comparison.

Cemi-SPA is a multicenter, retrospective, observational study conducted across 21 academic centers in Spain. Eligible patients were adults with histologically confirmed advanced or metastatic NSCLC and a PD-L1 expression level of ≥50% (as assessed by TPS), who received at least one dose of commercial cemiplimab monotherapy (350 mg every three weeks) as first-line treatment, with therapy initiation occurring no later than 30 September 2023. Patients with prior systemic therapy for early-stage NSCLC were eligible if treatment had been completed ≥6 months before the initiation of cemiplimab. Thus, out of an initial cohort of 162 patients, 150 were ultimately eligible for inclusion in the study (Figure 1).

The study was coordinated by Hospital 12 de Octubre, which obtained initial approval from its institutional review board (IRB) (Approval Number: 23/529). Next, invited Spanish collaborating centers obtained approval from their respective IRBs. Data were collected at each site using a standardized electronic case report form (eCRF) developed by the study team. All information was de-identified and stored on a password-protected server at Hospital 12 de Octubre, with no physical copies retained. This study adhered to the principles outlined in the Declaration of Helsinki and the WHO Guidelines for Good Clinical Practice. Owing to the retrospective nature of the study and the use of anonymized data, the requirement for written informed consent was waived by the participating IRBs. A full list of contributing institutions is provided in Supplementary Table S1.

To compare the real-world performance of cemiplimab, we also analyzed a historical control cohort approved by the Hospital 12 de Octubre Ethics Committee (Approval Number: 20/181). This cohort included patients with advanced NSCLC and PD-L1 TPS ≥ 50% who had received at least one dose of first-line single-agent pembrolizumab (either 2 mg/kg or 200 mg every three weeks). Pembrolizumab treatment in this cohort occurred between 2014 and 2022 at up to 10 academic hospitals within the Comunidad de Madrid. Participating institutions are listed in Supplementary Table S2.

### 2.2. Study Variables and Assessment

The primary aim of the study was to evaluate the safety, efficacy, and activity of single-agent cemiplimab in treatment-naïve patients with advanced NSCLC in a real-world setting. Secondary objectives included the identification of clinical risk factors associated with treatment outcomes. To reach that purpose, we first reviewed patient demographic characteristics, in addition to clinical characteristics available in the datasets, including PS-ECOG, smoking history, disease stage (according to the 8^th^ edition of the AJCC TNM classification), metastases allocation, histological features, PD-L1 expression, and results from genomic testing when available.

Radiological response was assessed by investigators at each center according to Response Evaluation Criteria In Solid Tumors (RECIST) without centralized adjudication. We defined overall response rate (ORR) as the rate of patients experiencing partial response or complete response as the best response. Regarding time-to-event analyses, we calculated OS and PFS from the treatment start date to the date of death or loss of follow-up, or the date of disease progression, death, or loss of follow-up, respectively. For those patients whose progression or death was not documented during the study period, the outcome was considered right-censored.

We collected treatment-related adverse events (TRAEs) that occurred during treatment with cemiplimab as defined by investigators and reported in clinical practice at the participating centers. We recorded TRAEs graded according to the National Cancer Institute Common Toxicity Criteria for Adverse Events (CTCAE; version 5.0), and we reported the cumulative crude incidence and maximum grade per patient.

In contrast, we extracted data from the historical pembrolizumab control cohort for comparison purposes. Among the collected variables, we selected clinical and demographic parameters, as well as efficacy and toxicity outcomes related to anti-PD-1 therapy, that were relevant for comparison with the findings of the Cemi-Spa study.

### 2.3. Statistical Considerations

We employed descriptive statistics to summarize baseline demographic and clinical characteristics, as well as treatment outcomes. For time-to-event analyses, including OS and PFS, we applied the Kaplan–Meier method and compared survival curves using the log-rank test. Hazard ratios (HRs) and 95% confidence intervals (Cis) were estimated using Cox proportional hazards regression models. Variables with a *p*-value < 0.05 in the univariate analysis for each survival outcome were included in the multivariate models. For categorical variables, we used the chi-squared (χ^2^) test or Fisher’s exact test, as appropriate.

To enhance the robustness of the comparative efficacy analysis between the cemiplimab and pembrolizumab cohorts, we performed a propensity score matching (PSM) analysis. We used this method to reduce potential confounding and adjust for imbalances in baseline characteristics across treatment groups. Matching was carried out using a nearest-neighbor algorithm without replacement in a 1:1 ratio, based on relevant clinical and demographic covariates.

All statistical tests were two-sided, and *p*-values < 0.05 were considered statistically significant. Given the retrospective nature of the study, no formal sample size calculation was conducted. All statistical analyses were performed using SPSS Statistics for Windows, Version 23.0 (IBM Corp., Armonk, NY, USA) and R version 4.4.3 for figures.

## 3. Results

### 3.1. Accrual and Patient Characteristics

- Cemi-SPA cohort.

We included a total of 150 patients with advanced NSCLC who initiated single-agent cemiplimab between 1 May 2022 and 30 September 2023. The baseline characteristics are provided in Table 1. The median age at treatment initiation was 70 years (range: 43–88 years), with the majority of patients being male (81.3%) and having a history of tobacco exposure (94.6%). Most patients presented with good performance status (PS-ECOG = 0–1, 85.3%). Adenocarcinoma histology was the most common subtype (69.1%), and most of the patients initiated cemiplimab at stage IV disease (88.0%). Next-generation sequencing (NGS) molecular aberration detection strategy was implemented in 43.3% of cases; among these, KRAS mutations were the most common molecular aberration detected (19.3%).

b. Pembrolizumab control cohort.

For comparison and analysis purposes, we included a historical control cohort with an alternative anti-PD-1 inhibitor. This cohort comprised 144 patients with advanced NSCLC who initiated first-line pembrolizumab monotherapy in a non-curative setting between 2014 and 2022. Key clinical characteristics of this cohort are detailed in Supplementary Table S3.

c. Differences among cohorts.

The baseline characteristics of patients were generally well balanced between the two cohorts (Table 2A), with two exceptions. (1) a higher proportion of patients with good ECOG-PS in the Cemi-SPA cohort (*p* = 0.042), (2) more frequent use of antibiotics within 30 days prior to the initiation of immunotherapy in the pembrolizumab cohort (*p* = 0.008).

### 3.2. Efficacy

a Cemi-Spa cohort.

**Figure 2:**
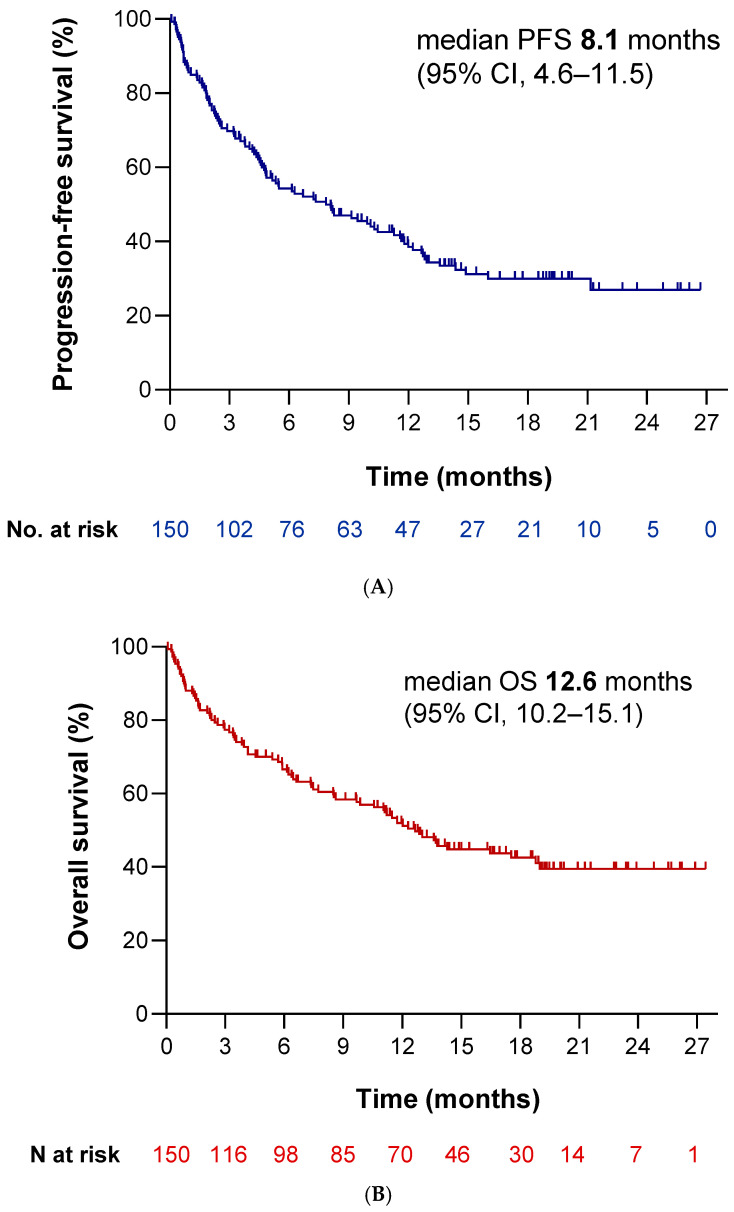

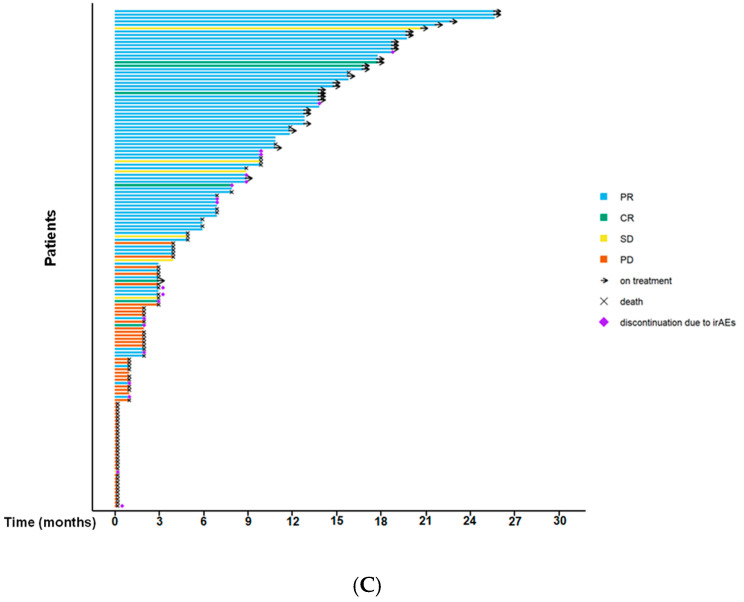
Outcomes for 150 patients with NSCLC treated in the cemiplimab cohort. (**A**) Kaplan–Meier curve for progression-free survival (PFS) for all 150 patients; mPFS: median PFS. (**B**) Kaplan–Meier curve for overall survival (OS) for all 150 patients; mOS: median OS. (**C**) Swimmer plot with radiographic response to cemiplimab by RECIST; CR: complete response, PR: partial response, SD: stable disease; PD: progressive disease.

With a median follow-up of 17.8 months (95% CI: 16.2–19.4 months), patients receiving cemiplimab achieved a median PFS time of 8.1 months (95% CI: 4.6–11.5 months) and a median OS time of 12.6 months (95% CI: 10.2–15.1 months) (Figure 2A and Figure 2B, respectively). Over half of the patients (56.9%) experienced a radiological response, with complete responses observed in 4.8% and partial responses in 52.1% (Figure 2C). The disease control rate (DCR) was achieved in 61.7% of patients (Table 3). The response rate across participating sites ranged from 0% to 100% (Supplementary Table S4). When excluding centers with ≤2 patients, no statistically significant differences were observed between sites (χ^2^ ≈ 15.7; *p* ≈ 0.39).

**Figure 3:**
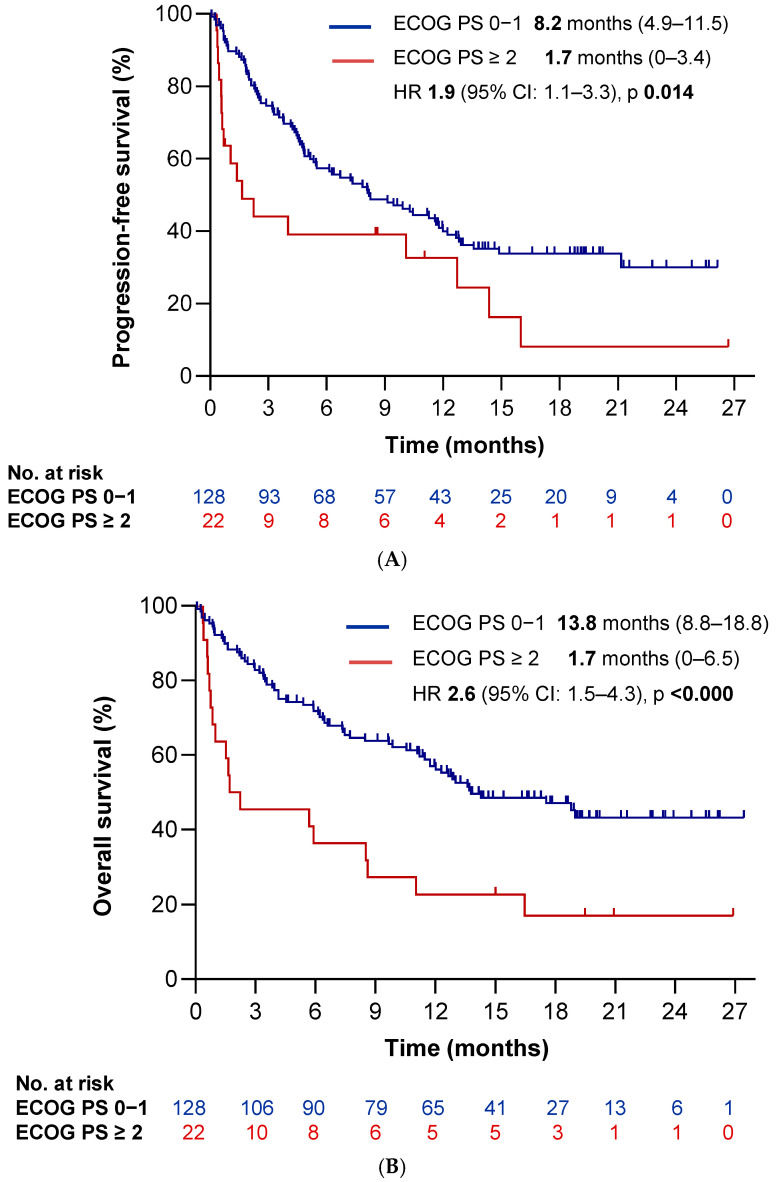
Survival outcomes according to ECOG performance status (PS). Kaplan–Meier curves illustrating (**A**) progression-free survival (PFS) and (**B**) overall survival (OS) stratified by ECOG-PS. The blue line corresponds to patients with ECOG PS 0–1, and the red line represents those with ECOG PS ≥ 2.

**Figure 4:**
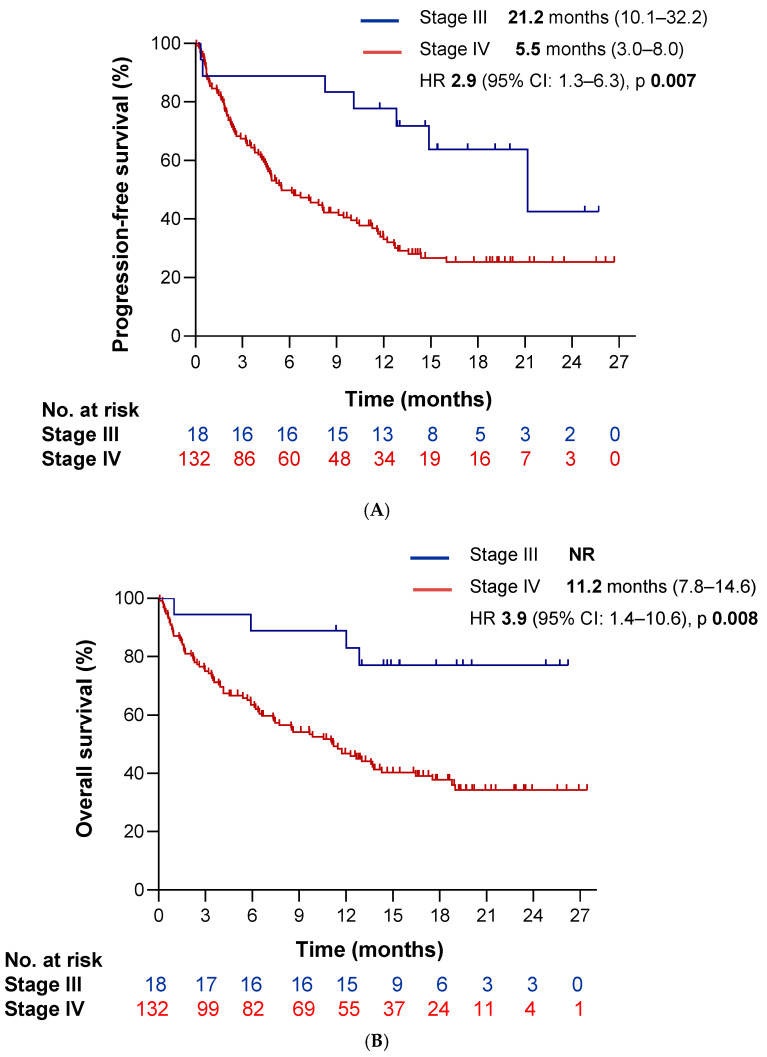
Survival outcomes according to disease stage. Kaplan–Meier curves illustrating (**A**) progression-free survival (PFS) and (**B**) overall survival (OS) stratified by disease stage at baseline. The blue line corresponds to patients with stage III NSCLC, and the red line represents those with stage IV disease.

To evaluate the impact of clinical variables on survival outcomes, we conducted univariate Kaplan–Meier survival analyses. Poor performance status (PS-ECOG ≥ 2) and stage IV disease were associated with significantly reduced PFS (HR 1.9 [95%CI 1.1–3.3], *p* = 0.014 and HR 2.9 [95%CI 1.3–6.3], *p* = 0.007, respectively) (Figure 3A and Figure 4A). Furthermore, the presence of extrathoracic disease, bone metastases, liver metastases, pleural and/or pericardial effusion, hypoalbuminemia, and >3 metastatic sites correlated significantly with worse PFS as well (HR 1.9 [95%CI 1.2–2.9], *p* = 0.004; HR 1.6 [95%CI 1.0–2.4], *p* = 0.042; HR 2.4 [95%CI 1.4–4.1], *p* = 0.002; HR 1.6 [95%CI 1.0–2.7], *p* = 0.045; HR 1.6 [95%CI 1.0–2.6], *p* = 0.042; and HR 1.9 [95%CI 1.2–3.0], *p* = 0.008, respectively). We observed similar associations for OS: ECOG ≥ 2 (HR 2.6 [95%CI 1.5–4.3], *p* < 0.0001), stage IV disease (HR 3.9 [95%CI 1.4–10.6], *p* = 0.008) (Figure 3B and Figure 4B), extrathoracic spread (HR 2.2 [95%CI 1.4–3.5], *p* = 0.001), >3 metastatic sites (HR 2.1 [95%CI 1.3–3.5], *p* = 0.002), and involvement of liver (HR 2.0 [95%CI 1.1–3.6], *p* = 0.026) or bone (HR 1.6 [95%CI 1.0–2.6], *p* = 0.031). Additionally, we observed worse OS in patients with lymphadenopathy at baseline (HR 2.6 [95%CI 1.3–5.4], *p* = 0.010), baseline corticosteroid use (10 mg of prednisone) (HR 2.2 [95%CI 1.4–3.6], *p* = 0.001), brain metastases (HR 2.1 [95%CI 1.3–3.4], *p* = 0.003), lymphopenia (< 1000 lymphocytes) (HR 2.1 [95%CI 1.2–3.6], *p* = 0.006), a derived neutrophil-to-lymphocyte ratio (dNLR) ≥ 3 (HR 2.0 [95%CI 1.3–3.0], *p* = 0.002), hypoalbuminemia (HR 1.8 [95%CI 1.1–3.0], *p* = 0.012), and a poor LIPI score (HR 1.5 [95%CI 1.0–2.4], *p* = 0.049). Conversely, the occurrence of a single-site metastasis was significantly associated with a more favorable PFS (HR 0.6 [95%CI 0.4–1.0], *p* = 0.041). The complete list of clinical variables are summarized in Table 4 and Table 5.

In the multivariate analysis, poor performance status (ECOG ≥ 2) emerged as the only variable significantly associated with both poorer PFS (HR 1.78 [95%CI 1.01–3.09], *p* = 0.044) and OS (HR 2.6 [95%CI 1.50–4.56], *p* = 0.001) (Table 6A,B).

**Figure 5:**
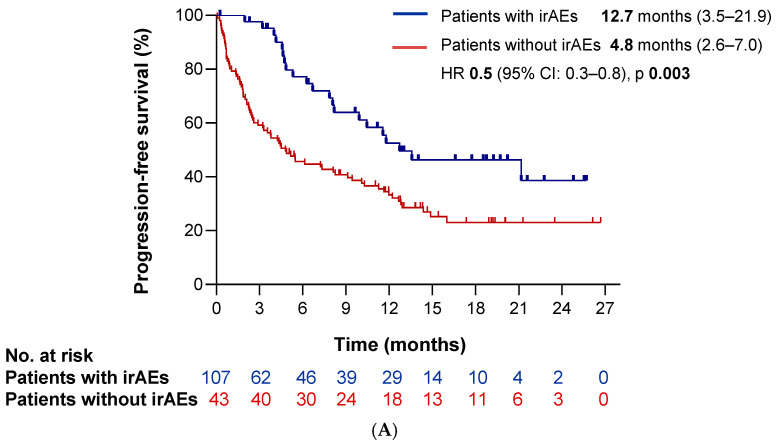

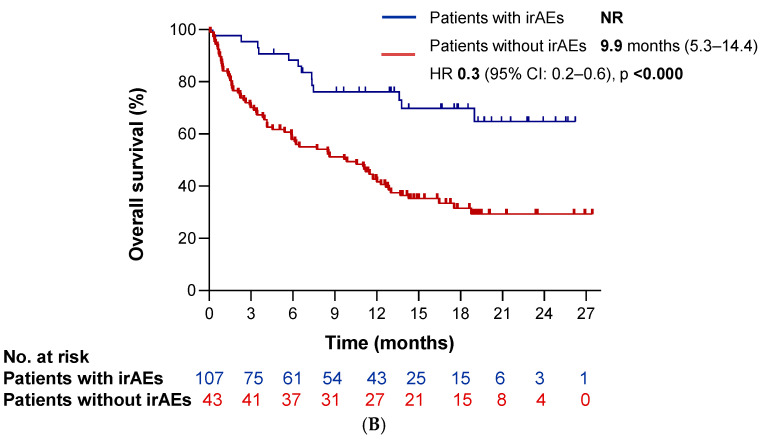
Survival outcomes based on the occurrence of immune-related adverse events (irAEs). Kaplan–Meier curves showing (**A**) progression-free survival (PFS) and (**B**) overall survival (OS) according to the development of irAEs. The blue line represents patients who experienced irAEs, while the red line corresponds to those who did not.

We also investigated whether the occurrence of immune-related adverse events (irAEs) was associated with clinical outcomes. Notably, the development of irAEs was significantly associated with improved survival. Patients experiencing irAEs had better PFS (HR 0.5 [95%CI 0.3–0.8], *p* = 0.003) and OS (HR 0.3 [95%CI 0.2–0.6], *p* < 0.0001) compared to those without irAEs (Figure 5A,B).

b Pembrolizumab historical cohort and comparison between both cohorts.

**Figure 6:**
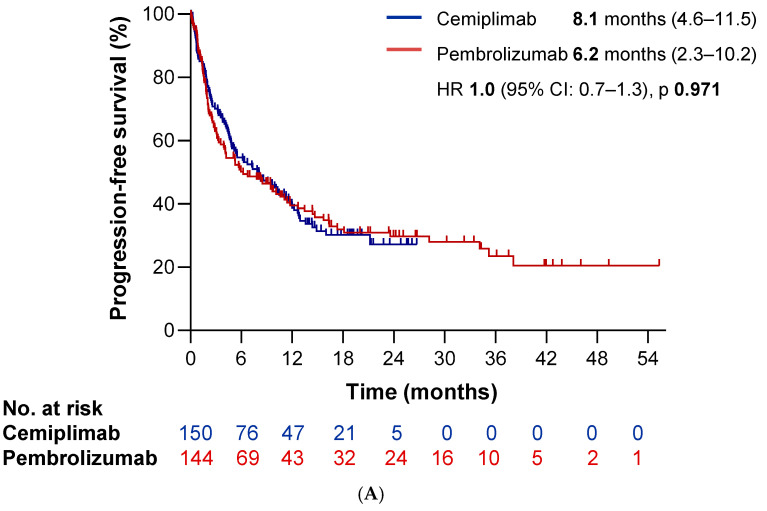

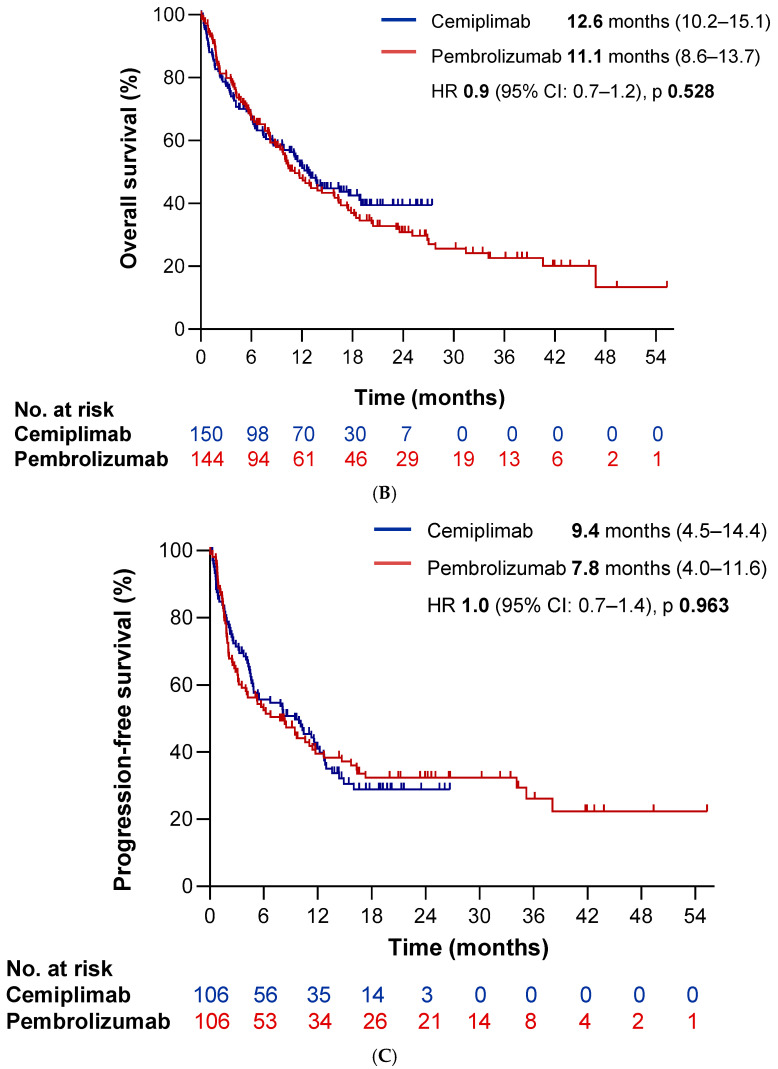

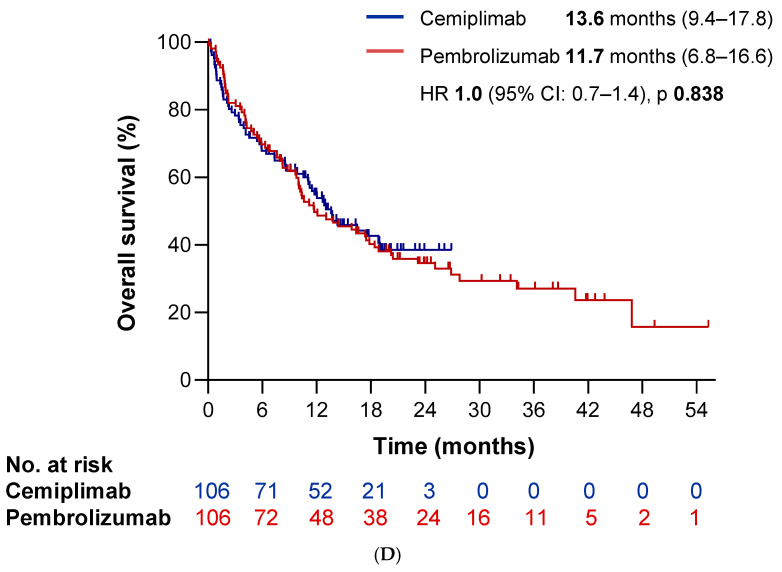
Survival outcomes comparing cemiplimab and pembrolizumab cohorts. Kaplan–Meier curves for (**A**) progression-free survival (PFS) and (**B**) overall survival (OS) in the unmatched cohorts: patients treated with cemiplimab (blue) vs. pembrolizumab (red). Panels (**C**,**D**) depict PFS and OS, respectively, after applying propensity score matching (PSM).

To confirm the real-world efficacy findings of cemiplimab and explore potential differences in efficacy among anti-PD-1 blockers, we compared the Cemi-SPA cohort with the pembrolizumab cohort. With a median follow-up time of 30.2 months, the pembrolizumab cohort achieved a median PFS time of 6.2 months (95% CI: 2.3–10.2 months) and a median OS time of 11.2 months (IC 95% 8.6–13.7 months), With no statistically significant differences between the two cohorts (HR 0.99 *p* = 0.97 and HR 0.91 *p* = 0.53, respectively; (Figure 6A,B). Additionally, while no significant differences were observed in terms of DCR (*p* = 0.4), we identified a statistically significant difference in ORR, which was higher in the cemiplimab cohort compared to the pembrolizumab cohort, 56.9% vs. 44.5% (Odds Ratio 1.65, *p* = 0.046). Supplementary Tables S5 and S6 show survival outcomes according to clinical baseline characteristics.

In the matched population, efficacy outcomes remained comparable with no statistically significant differences in terms of survival outcomes or radiological response. The median PFS time was 9.4 months (95% CI: 4.5–14.4 months) in the cemiplimab cohort versus 7.8 months (95% CI: 4.0–11.6 months) in the pembrolizumab cohort (HR 1.01, *p* = 0.96). Similarly, median OS time was 13.6 months (95% CI: 9.4–17.8 months) and 11.7 months (95% CI: 6.8–16.6 months), respectively (HR 0.96, *p* = 0.84) (Figure 6C,D). Finally, ORR was numerically higher with cemiplimab (58.3% vs. 46.2%), though not statistically significant (Odds Ratio 1.62, *p* = 0.097).

### 3.3. Safety of Cemi-Spa Cohort

Among the 150 patients analyzed, irAEs of any grade occurred in 43 (28.7%) cases. The majority of these irAEs were mild to moderate in severity (grade 1–2 in 16.7% patients), while 18 patients (12.0%) experienced grade ≥ 3 irAEs. The most frequently reported irAEs were hepatitis and pneumonitis, each occurring in 9 patients (6.0%), with 5 cases (3.3%) classified as grade ≥ 3. Less common irAEs observed were colitis (3.3%) and hypo-/hyperthyroidism (0.7%). Notably, irAEs led to treatment discontinuation in 20 patients (13.3%), and one patient (0.7%) experienced a fatal irAE.

Regarding clinical variables, we observed a significant association between the occurrence of irAEs and good ECOG PS 0–1 (*p* = 0.039). Nevertheless, despite this association, the development of irAEs remained an independent prognostic factor for both PFS and OS after adjusting for ECOG PS (HR 0.51, *p* = 0.006 for PFS; HR 0.47, *p* = 0.003 for OS). Safety results are listed in Table 7.

## 4. Discussion

To the best of our knowledge, this is the first study reporting real-world data on cemiplimab in a cohort of patients with advanced NSCLC and PD-L1 expression ≥ 50%. We included 150 real-life patients treated with single-agent cemiplimab, with a median follow-up time of 17.8 months. We observed a median PFS time and median OS time of 8.1 (95% CI: 4.6–11.5 months) and 12.6 months (95% CI: 10.2–15.1 months), respectively. Our findings align with most real-world studies evaluating other anti-PD-1 agents, as well as with our own historical control cohort treated with pembrolizumab.

In terms of overall efficacy, our results revealed a similar median PFS time compared with the pivotal EMPOWER-Lung 1 trial (7.9 months vs. 8.1 months in the experimental arm). However, we documented a markedly worse median OS time. We attribute these differences to the poorer baseline characteristics of our cohort compared to those in the clinical trial. Notably, our population included a higher proportion of patients with ECOG-PS ≥ 2 (14.7% vs. 0%), older median age (70 vs. 63 years), no never-smokers (5.5% vs. 0%), a higher percentage of brain metastases (18.7% vs. 12%), and increased tumor burden as 20.7% of patients having metastases in ≥3 sites and 28% presenting with bone metastases. Additionally, many patients had significant comorbidities [8,9].

Despite these adverse features, the survival curves suggested the presence of a tail effect, consistent with long-term survivors, as reported in clinical trials. Up to 44% of patients in our cohort were still alive at 17 months, reinforcing this impression. In this regard, a retrospective real-world study has reported a comparable proportion of long-term benefit with pembrolizumab, similar to that observed in the KEYNOTE-024 trial [10]. Due to the substantially shorter median follow-up in our cohort, we were not able to fully confirm these findings; however, the emerging tail effect supports this potential trend.

Similarly, our historical pembrolizumab cohort showed comparable efficacy outcomes in terms of both PFS and OS, with no statistically significant differences. Baseline characteristics were generally well balanced; however, patients treated with pembrolizumab more frequently presented with ECOG-PS ≥ 2 and prior antibiotic exposure. We believe these baseline imbalances may be partially explained by recent studies reporting a detrimental effect of antibiotic use on the efficacy of anti-PD-1 monotherapy, an effect that appears less pronounced when immunotherapy is combined with chemotherapy [11]. In addition, a marginal benefit of single-agent immunotherapy has been reported in patients with poor functional status [12]. These insights may have contributed to the current trend of reserving single-agent immunotherapy for patients with more favorable clinical characteristics. Finally, even after PSM to adjust for baseline differences, we did not identify any significant disparities in efficacy between the two cohorts, suggesting a comparable real-world performance of both anti-PD-1 agents. In contrast to our real-world findings, a recent network meta-analysis comparing first-line immune checkpoint inhibitor monotherapies in advanced NSCLC patients with PD-L1 expression ≥50% suggested that cemiplimab may offer superior efficacy over pembrolizumab in terms of PFS and ORR, with comparable OS and no significant differences in safety profiles [13]. Several factors may explain this apparent discrepancy. First, network meta-analyses are based on indirect comparisons across clinical trials that often differ in terms of patient eligibility criteria, study design, and follow-up duration. Second, pivotal trials enrolled highly selected populations with optimal performance status and limited comorbidities, which may not reflect the clinical heterogeneity of real-world practice. Additionally, other meta-analyses have reported no significant differences in efficacy among various anti-PD-1/PD-L1 agents, supporting the notion that these therapies may offer comparable benefits in the real-world setting [14]. Therefore, in the absence of direct head-to-head clinical trials and due to the heterogeneity of results supporting indirect comparisons, we believe that current evidence is insufficient to recommend one anti-PD-1 agent over another for this patient population.

The observed discrepancy in OS between our real-world cohort and the pivotal trial (12.6 vs. 26.1 months), despite similar PFS, mirrors findings from other real-world studies on anti-PD-1 monotherapy in this setting [15]. Most of these studies report PFS values comparable to ours, while medianOS time ranges widely from 15.2 to 25.5 months [16,17,18,19], likely reflecting differences in patient populations and treatment settings. Among the potential reasons explaining the discrepancies in OS outcomes between clinical trials and real-world studies, we highlight the higher burden of comorbidities and the more frequent prevalence of patients with poor baseline functional status (ECOG PS ≥ 2) in real-life cohorts. Additionally, a lower proportion of patients in real-world settings typically receive subsequent lines of anticancer therapy after progression, which may further contribute to inferior long-term survival outcomes when compared to trial populations.

We identified several clinical variables associated with poorer outcomes. Advanced disease stage, ECOG PS ≥ 2, liver and bone metastases, extrathoracic disease, and the presence of metastases in ≥ 3 anatomical sites were all associated with inferior PFS. These same variables, along with lymphopenia and poor LIPI score, were also linked to poorer OS. These results are consistent with previously published real-world data [20]. Contrary to our preliminary data and some prior reports, we did not observe significant associations between survival outcomes and antibiotic exposure or BMI [11,21]. Interestingly, a Spanish real-world study of pembrolizumab also failed to identify such associations [16], which may be due to differences in sample size, follow-up duration, or definitions of exposure windows.

Likewise, we did not find a significant association between smoking status and response to immunotherapy. Although some literature supports a potential association [22], this remains controversial, and existing meta-analyses show high heterogeneity [14,23]. Moreover, older datasets often lacked broad molecular profiling, potentially overlooking actionable driver alterations that are more prevalent among never-smokers (e.g., RET, MET, HER2), especially in the absence of NGS [24].

We also found no significant association between the level of PD-L1 expression and treatment efficacy, unlike the pivotal study [7]. We suspect that this discrepancy may stem from heterogeneity in PD-L1 assessment, sample quality issues in real-world practice, and the multicentric design of our study, which could have introduced variability in PD-L1 quantification. Additionally, KRAS mutation status did not appear to influence treatment efficacy outcomes in our cohort. This observation is consistent with previously published data, where the presence of KRAS mutations has been associated with preserved or even improved responsiveness to anti-PD-1 [25]. However, we acknowledge a major limitation in the lack of comprehensive molecular profiling, as only 33.3% of patients underwent NGS testing. In most participating centers, molecular testing was limited to common driver mutations (ALK, EGFR, ROS1, BRAF), and the heterogeneity of platforms used may have further constrained molecular analyses. Thus, conclusions regarding molecular correlates of response must be interpreted cautiously.

In our multivariate analysis, only ECOG performance status remained associated with both PFS and OS. Functional status clearly emerged as the most relevant prognostic factor. Consistent with previous studies, we observed a significant difference in survival between patients with ECOG 0–1 (median PFS time of 8.2 months 95% CI: 4.9–11.5 months and median OS time: 13.8 months 95% CI: 8.8–18.8 months) and those with ECOG ≥ 2 (median PFS time of 1.6 months 95% CI: 0–3.6 months and median OS time: 1.7 months 95% CI: 0–6.5 months).

A recent retrospective Italian study, including 153 patients with ECOG 2 and PD-L1 ≥ 50% NSCLC treated with pembrolizumab, also underscored the prognostic relevance of performance status. In that study, the origin of functional impairment—whether tumor-related or due to comorbidities—impacted survival outcomes significantly [26]. We did not collect data on the cause of ECOG PS deterioration in our cohort, which limits direct comparison. Nevertheless, our findings are supported by recent 5-year real-world data on pembrolizumab, which identified ECOG-PS as the primary determinant of long-term benefit (10).

Finally, we reported favorable safety outcomes in our real-world cohort. The incidence of any-grade irAEs was 28.7%, and grade ≥ 3 irAEs occurred in 12% of patients—figures lower than those reported in the pivotal trial (53% and 23%, respectively). This discrepancy likely reflects underreporting of adverse events in real-world settings. Interestingly, we observed a higher discontinuation rate because of toxicity in our cohort (13.3% vs. 7%), potentially reflecting less controlled clinical environments, increased patient vulnerability, and greater prevalence of autoimmune disorders [9]. In fact, one real-world study evaluating cemiplimab in cutaneous squamous cell carcinoma reported similar rates of grade ≥ 3 irAEs (18.5%) and treatment discontinuation (9.9%) [27]. As reported in prior trials and observational studies of immunotherapy, the occurrence of severe irAEs leading to treatment discontinuation was associated with improved survival in our cohort, suggesting a potential link between immune activation and treatment efficacy [28,29,30]. Although we observed a significant association between baseline ECOG-PS 0–1 and the development of irAEs, this effect remained statistically significant for both PFS and OS after adjusting for ECOG-PS, supporting the independent prognostic value of immune-related toxicity. However, the remarkably strong association identified in our analysis (HR 0.3) should be interpreted cautiously. Unfortunately, the onset of immune-related toxicities was not systematically recorded in our dataset, which prevents performing time-dependent analyses to fully address potential immortal time or detection biases. Consequently, the relationship between irAEs and improved survival might partly reflect this temporal interplay rather than a purely causal effect.

### Limitations

The results of this study should be considered in the context of a number of limitations. The retrospective nature of this study and the inherent challenges of real-world data collection may have introduced potential selection and information biases. For instance, the accuracy of the RECIST criteria application in real-world settings for evaluating immunotherapy response may have been variable among different investigators. Furthermore, given the multicenter design, PD-L1 assessment via immunohistochemistry was performed by multiple pathologists, potentially introducing interobserver variability in the semi-quantitative nature of this technique.

The absence of comprehensive molecular profiling, particularly NGS, in a substantial proportion of patients (66.6%) represents another limitation. In many centers, molecular testing was restricted to a limited panel of genes, such as ALK, EGFR, ROS1, and BRAF. Consequently, the interplay between molecular alterations and response to cemiplimab could not be fully assessed in a significant portion of the cohort. Additionally, the heterogeneity of NGS panels (commercial or in-house) employed across different centers may have influenced the interpretation of molecular findings. Therefore, conclusions drawn regarding molecular correlates of response should be interpreted with caution.

Finally, the study did not systematically capture information on subsequent lines of therapy, which may have influenced overall survival outcomes. Moreover, due to the more recent treatment window of the cemiplimab cohort, the follow-up period was shorter compared with the pembrolizumab group, potentially limiting the ability to identify long-term survival differences.

## 5. Conclusions

Cemi-SPA RWD on cemiplimab further corroborated the favorable safety profile and efficacy outcomes observed in pivotal clinical trials. The consistency of our findings with other RWD of anti-PD-1/PD-L1 agents reinforces the robust nature of these therapeutic agents in clinical practice and provides valuable insights into the clinical application of cemiplimab beyond the confines of controlled clinical trials. We identified several clinical variables associated with survival outcomes, most of which have been previously reported in the literature. Among all clinical and pathological variables analyzed, PS-ECOG emerged as the most decisive factor influencing survival outcomes. In the multivariate model, a poor functional status (PS-ECOG ≥ 2) remained independently associated with both reduced PFS and OS, underscoring its prognostic relevance in patients receiving first-line cemiplimab. Conversely, the development of irAEs emerged as a strong prognostic factor, significantly associated with improved survival outcomes. Our findings support the use of cemiplimab as an effective therapeutic option in routine clinical practice and reinforce the prognostic relevance of baseline clinical variables and on-treatment biomarkers. Moreover, there remains a clear need for prospective real-world studies to further elucidate the clinical utility of cemiplimab in this setting and to identify robust predictive biomarkers that can guide individualized treatment strategies.

## Figures and Tables

**Table 1:** Patient characteristics at baseline in Cemiplimab cohort (n = 150).

Variable	n (%)
**Age at diagnosis (range 43–88)**	Mean 70, SD 10.2 (67.3% > 65 years)
**Sex**	
Male	122 (81.3)
Female	28 (18.7)
**Smoking status**	
History of smoking	142 (94.6)
Never smoker	8 (5.4)
**Smoking pack years > 30**	72 (48.0)
**ECOG PS**	
0–1	128 (85.3)
≥2	22 (14.7)
**BMI**	
<18	13 (9.7)
18–25	54 (40.3)
25–30	48 (35.8)
>30	19 (14.2)
**Histology**	
Adenocarcinoma	103 (69.1)
Squamous	34 (22.8)
NOS	13 (8.1)
**PD-L1 expression**	
≥50–<60%	42 (28.0)
≥60–<90%	69 (46.0)
≥90%	38 (25.3)
Not specified	1 (0.7)
**Molecular alterations**	
KRAS	29 (19.3)
Other than KRAS	18 (12.0)
**Extrathoracic disease**	
Yes	90 (60.8)
No	
**Disease stage**	
III	18 (12.0)
IV	132 (88.0)
**Number of metastatic sites**	
1	46 (30.7)
2	44 (29.3)
≥3	31 (20.7)
**Malignant pleural/pericardial effusion**	
Yes	27 (18.0)
No	123 (82.0)
**CNS metastases**	
Yes	28 (18.7)
No	122 (81.3)
**Liver metastases**	
Yes	17 (11.3)
No	133 (88.7)
**Bone metastases**	
Yes	42 (28.0)
No	108 (72.0)
**Adenopathies**	
Yes	125 (83.3)
No	25 (16.7)
**Steroids administration (>10 mg of prednisone)**	
Yes	31 (20.7)
No	119 (79.3)
**Prior Antibiotics**	
Yes	33 (22.1)
No	117 (77.9)
**Hypoalbuminemia**	
Yes	36 (26.1)
No	114 (73.9)
**LIPI score**	
Good	55 (40.1)
Intermediate	59 (39.3)
Poor	23 (16.8)
**Lymphopenia (<1000 lymphocytes)**	
Yes	21 (14.3)
No	129 (85.7)
**dNLR ≥ 3**	
Yes	50 (33.3)
No	100 (66.6)
**LDH > ULN**	
Yes	55 (40.1)
No	95 (59.9)
**Comorbidities**	
Hypertension	88 (58.7)
Dyslipidemia	67 (44.7)
Diabetes mellitus	37 (24.7)
Cardiovascular disease	30 (20.0)
HIV infection	2 (1.3)
HBV/HCV infection	14 (9.3)
**Previous anticancer therapies**	
Surgery	10 (6.7)
Radiotherapy (Definitive)	11 (7.3)
Radiotherapy (Palliative)	22 (14.7)
Chemotherapy (Neo/adjuvant)	7 (4.6)
Chemotherapy + Radiotherapy	3 (2.0)

BMI: Body Mass Index; CNS: Central Nervous System; dNLR: Derived Neutrophil-to-Lymphocyte Ratio; ECOG PS: Eastern Cooperative Oncology Group Performance Status; LIPI: Lung Immune Prognostic Index; SD: Standard Deviation; ULN: Upper Limit of Normal.

**Table 2:** (**A**) Clinical characteristics differences among cohorts. (**B**) Clinical characteristics differences among cohorts after PMS (N = 106 p/group).

(**A**)			
**Variable**	**Cemiplimab Cohort** **n 150 (%)**	**Pembrolizumab Cohort n 144 (%)**	***p* Value**
**Age**			0.70
<65years	52 (34.7)	53 (36.8)	
≥65years	98 (65.3)	91 (63.2)	
**Sex**			0.60
Male	122 (81.3)	108 (75.0)	
Female	28 (18.7)	36 (25.0)	
**ECOG-PS at beginning IT treatment**			* 0.042
0	32 (25.0)	25 (24.0)	
1	96 (83.5)	79 (68.1)	
2	19 (86.4)	37 (92.5)	
3	3 (13.6)	3 (7.5)	
**Smoking status**			0.57
Never	8 (5.4)	5 (3.5)	
Prior history of tobacco	141 (94.6)	139 (96.5)	
**Histology**			0.19
Non-squamous	115 (77.2)	101 (70.1)	
Squamous	34 (22.8)	43 (29.9)	
**PD-L1 expression**			0.63
≥50–<60%	44 (39.6)	38 (38.8)	
>60–≤90%	67 (63.8)	60 (58.3)	
≥90%	38 (36.2)	43 (41.7)	
**Stage at the beginning of immunotherapy**			0.14
III	18 (12.0)	10 (6.9)	
IV	132 (88.0)	134 (93.1)	
**Antibiotics**			0.01 *
Yes	33 (22.1)	52 (36.1)	
No	116 (77.9)	92 (63.9)	
**Liver metastases**			0.49
Yes	17 (11.3)	21 (14.6)	
No	133 (88.7)	123 (85.4)	
**CNS metastases**			1
Yes	28 (18.7)	27 (18.8)	
No	122 (81.3)	117 (81.2)	
**LIPI Score**			0.25
Good	55 (48.2)	38 (40.9)	
Intermediate	59 (72.0)	55 (66.3)	
Poor	23 (28.0)	28 (33.7)	
**dNLR ≥ 3**			0.23
Yes	50 (34.0)	59 (41.0)	
No	97 (66.0)	85 (59.0)	
**LDH > ULN**			0.17
Yes	55 (40.1)	59 (48.8)	
No	82 (59.9)	62 (51.2)	
(**B**)			
**Characteristic**	**Cemiplimab,** **n 106(%)**	**Pembrolizumab,** **n 106 (%)**	***p* Value**
**Age**			1
<65years	40 (37.7)	39 (36.8)	
≥65years	66 (62.3)	67 (63.2)	
**Sex**			1
Male	82 (77.4)	77 (77.8)	
Female	24 (22.6)	22 (22.2)	
**ECOG-PS at beginning IT treatment**			0.95
0	23 (21.7)	23 (21.7)	
1	64 (60.4)	67 (63.2)	
2	17 (16.0)	14 (13.2)	
3	2 (1.9)	2 (1.9)	
**Smoking status**			0.06
Never smoker	8 (7.6)	2 (1.9)	
Prior history of tobacco	97 (92.4)	104 (98.1)	
**Histology**			1
Non-squamous	75 (70.8)	76 (71.7)	
Squamous	31 (29.2)	30 (28.3)	
**PD-L1 expression**			0.12
50–≤60	36	27	
>60–<90	46	42	
≥90	23	36	
**Stage**			1
Stage III	9 (8.5)	9 (8.5)	
Stage IV	97 (91.5)	97 (91.5)	
**Antibiotics**			0.77
No	75 (70.8)	72 (67.9)	
Yes	31 (29.2)	34 (32.1)	
**Liver metastases**			1
No	94 (88.7)	94 (88.7)	
Yes	12 (11.3)	12 (11.3)	
**CNS metastases**			0.86
No	87 (82.1)	89 (84.0)	
Yes	19 (17.9)	17 (16.0)	
**LIPI Score**			0.22
Good	41 (42.7)	27 (30.7)	
Intermediate	40 (41.7)	42 (47.7)	
Poor	15 (15.6)	19 (21.6)	
**dNLR**			0.15
Normal	74 (71.2)	65 (61.3)	
High	30 (28.8)	41 (38.7)	
**LDH**			0.3
Normal	56 (58.3)	44 (50.0)	
High	40 (41.7)	44 (50.0)	

CNS: Central Nervous System; dNLR: Derived Neutrophil-to-Lymphocyte Ratio; ECOG PS: Eastern Cooperative Oncology Group Performance Status; LIPI: Lung Immune Prognostic Index; ULN: Upper Limit of Normal; * = clinically significant.

**Table 3:** Tumor response to Cemiplimab.

Response	Evaluable Patients (n = 150)
**Overall response rate, %**	56.9
**Disease control rate, %**	61.7
**Best overall response, n (%)**	
Complete response	7 (4.8)
Partial response	76 (52.1)
Stable disease	7 (4.8)
Progressive disease	56 (38.4)

**Table 4:** Univariate analysis for PFS in patients treated with cemiplimab.

Variable	Median PFS (95% CI, Months)	HR	IC 95% (Lower–Upper)	*p*-Value
**Disease stage**		2.9	(1.3–6.3)	0.007 *
III	21.2 (10.1–32.2)			
IV	5.5 (3.0–8.0)			
**Liver metastases**		2.4	(1.4–4.1)	0.002 *
Yes	3.2 (0–6.6)			
No	9.4 (5.6–13.3)			
**ECOG PS**		1.9	(1.1–3.3)	0.014 *
0–1	8.2 (4.9–11.5)			
≥2	1.7 (0–3.4)			
**Extrathoracic disease**		1.9	(1.2–2.9)	0.004 *
Yes	4.8 (2.7–6.9)			
No	12.8 (10.0–15.6)			
**≥3 metastatic sites**		1.9	(1.2–3.0)	0.008 *
Yes	2.6 (1.0–4.2)			
No	10.1 (6.9–13.2)			
**Bone metastases**		1.6	(1.0–2.4)	0.037 *
Yes	4.0 (2.3–5.7)			
No	10.1 (6.9–13.3)			
**Hypoalbuminemia**		1.6	(1.0─2.6)	0.042 *
Yes	5.3 (1.3–9.3)			
No	10.4 (6.3–14.6)			
**Pleural/pericardial effusion**		1.6	(1.0–2.7)	0.045 *
Yes	4.8 (2.6–7.0)			
No	9.4 (5.9–13.0)			
**LIPI score**		1.4	(1.0–1.9)	0.032 *
Good	11.6 (6.2–17.1)			
Intermediate	8.2 (2.0–14.4)			
Poor	2.5 (1.4–3.5)			
**Single-site metastases**		0.6	(0.4–1.0)	0.041 *
Yes	12.0 (9.5–14.4)			
No	5.5 (3.2–7.7)			
**IrAEs**		0.5	(0.3–0.8)	0.003 *
Yes	12.7 (3.5–22.0)			
No	4.8 (2.6–7.0)			
**CNS metastases**		1.5	(0.9–2.5)	0.106
Yes	3.8 (0–8.9)			
No	9.1 (5.7–12.5)			
**Adenopathies**		1.4	(0.8–2.5)	0.204
Yes	7.2 (4.4–10.1)			
No	12.2 (6.8–17.7)			
**Baseline steroids**		1.4	(0.8–2.3)	0.194
Yes	3.5 (0.5–6.6)			
No	8.2 (5.1–11.4)			
**LDH > ULN**		1.4	0.9–2.1	0.122
Yes	6.1 (2.3–10.0)			
No	10.1 (4.9–15.3)			
**Lymphopenia (<1000 lymphocytes)**		1.3	(0.7–2.3)	0.372
Yes	4.0 (0.6–7.4)			
No	8.2 (4.8–11.6)			
**Prior antibiotics**		1.3	0.8–2.1	0.245
Yes	4.3 (2.6–6.1)			
No	8.2 (4.5–11.8)			
**dNLR ≥ 3**		1.3	0.9–2.0	0.170
Yes	4.3 (1.5–7.2)			
No	9.9 (6.6–13.3)			
**KRAS mutations**		1.1	0.7–2.0	0.645
Yes	8.1 (1.4–14.8)			
No	7.2 (4.0–10.5)			
**Histology**		1.0	0.6–1.7	0.861
Non-squamous	8.1 (4.3–11.9)			
Squamous	7.3 (2.5–12.1)			
**Sex**		0.9	(0.6–1.6)	0.836
Male	7.3 (3.6–11.0)			
Female	8.1 (0–20.5)			
**PD-L1 ≥ 90%**		0.7	(0.4–1.1)	0.109
Yes	11.6 (5.0–18.3)			
No	7.2 (4.2–10.3)			
**Smoking history**		1.5	(0.5–4.0)	0.460
Never smoker	NR			
Former smoker	7.3 (2.6–12.0)			
Current smoker	8.2 (3.2–13.2)			
**Age**		0.8	(0.5–1.2)	0.307
<65 years	4.8 (0–10.0)			
≥65 years	9.4 (5.4–13.5)			

CI: Confidence Interval; CNS: Central Nervous System; dNLR, derived neutrophil-to-lymphocyte ratio; ECOG PS: Eastern Cooperative Oncology Group Performance Status; irAEs: immune-related adverse events; LIPI, lung immune prognostic index; NR: not reached; PFS: progression-free survival; ULN: Upper Limit of Normal; * Clinical significant.

**Table 5:** Univariate analysis for OS in patients treated with cemiplimab.

Variable	Median OS (95% CI, Months)	HR	IC 95% (Lower–Upper)	*p*-Value
**Disease stage**		3.9	(1.4–10.6)	0.008 *
III	NR			
IV	11.2 (7.8–14.6)			
**Liver metastases**		2.0	(1.1–3.6)	0.026 *
Yes	7.4 (5.8–8.9)			
No	13.6 (8.5–18.7)			
**ECOG PS**		2.6	(1.5–4.3)	<0.000 *
0–1	13.8 (8.8–18.8)			
≥2	1.7 (0–6.5)			
**Extrathoracic disease**		2.2	(1.4–3.5)	0.001 *
Yes	7.4 (4.2–10.5)			
No	NR			
**≥3 metastatic sites**		2.1	(1.3–3.5)	0.002 *
Yes	4.5 (0.7–8.4)			
No	NR			
**Bone metastases**		1.6	(1.0–2.6)	0.031 *
Yes	5.9 (1.2–10.6)			
No	13.8 (8.3–19.3)			
**Hypoalbuminemia**		1.8	(1.1–3.0)	0.012 *
Yes	6.6 (1.6–11.6)			
No	NR			
**Pleural/pericardial effusion**		1.6	(1.0–2.8)	0.062
Yes	9.7 (0.5–18.8)			
No	13.7 (7.5–19.9)			
**LIPI score**		1.5	(1.0–2.4)	0.049 *
Good	NR			
Intermediate	12.8 (10.1–15.6)			
Poor	3.2 (1.4–4.9)			
**Single-site metastases**		0.7	(0.4–1.1)	0.100
Yes	NR			
No	11.2 (7.7–14.7)			
**IrAEs**		0.3	(0.2–0.6)	<0.000 *
Yes	NR			
No	9.9 (5.3–14.4)			
**CNS metastases**		2.1	(1.3–3.4)	0.003 *
Yes	4.5 (0–9.6)			
No	13.8 (8.1–19.5)			
**Adenophaties**		2.6	(1.3–5.4)	0.010 *
Yes	11.0 (7.6–14.5)			
No	NR			
**Baseline steroids**		2.2	(1.4–3.6)	0.001 *
Yes	2.9 (0–8.0)			
No	16.5 (11.4–21.5)			
**LDH > ULN**		1.5	(1.0–2.4)	0.080
Yes	8.6 (1.5–15.8)			
No	17.5 (10.9–24.2)			
**Lymphopenia (<1000 lymphocytes)**		2.1	(1.2–3.6)	0.006 *
Yes	4.1 (0–9.0)			
No	13.8 (8.5–19.0)			
**Prior antibiotics**		1.6	(1.0–2.5)	0.071
Yes	8.5 (2.5–14.4)			
No	13.7 (8.0–19.4)			
**dNLR ≥ 3**		2.0	(1.3–3.0)	0.002 *
Yes	6.4 (4.5–8.3)			
No	NR			
**KRAS mutations**		1.1	(0.6–2.0)	0.687
Yes	12.6 (8.1–17.2)			
No	13.6 (7.7–19.6)			
**Histology**		1.2	(0.7–2.0)	0.441
Non-squamous	13.7 (8.1–19.3)			
Squamous	11.0 (5.9–16.2)			
**Sex**		0.9	(0.5–1.6)	0.757
Male	12.3 (8.9–15.7)			
Female	12.8 (2.9–22.8)			
**PD-L1 ≥ 90%**		0.8	(0.5–1.3)	0.310
Yes	NR			
No	12.3 (9.7–14.9)			
**Smoking history**		0.9	(0.4–2.2)	0.810
Never smoker	5.7 (0–17.2)			
Former smoker	12.3 (7.1–17.5)			
Current smoker	13.0 (6.9–17.5)			
**Age ≥ 65 years**		0.8	(0.5–1.3)	0.424
<65 years	11.7 (3.8–19.6)			
≥65 years	12.8 (8.0–17.7)			

CI: Confidence Interval; CNS: Central Nervous System; dNLR, derived neutrophil-to-lymphocyte ratio; ECOG PS: Eastern Cooperative Oncology Group Performance Status; irAEs: immune-related adverse events; LIPI, lung immune prognostic index; NR: not reached; OS: overall survival; ULN: Upper Limit of Normal; * clinically significant.

**Table 6:** (**A**) Cox Multivariate analysis for PFS. (**B**) Cox Multivariate analysis for OS.

(**A**)			
**Variable**	**HR**	**95% CI (Lower–Upper)**	***p*-Value**
ECOG-PS	1.78	1.01–3.09	0.045 *
Stage IV	2.00	0.80–5.01	0.140
Bone metastasis	1.09	0.62–1.91	0.767
Liver metastasis	1.62	0.81–3.25	0.170
Extrathoracic metastasis	1.30	0.72–2.37	0.386
>3 metastatic sites	1.06	0.56–2.02	0.851
Albumin at baseline	1.32	0.77–2.27	0.316
Age ≥ 65	0.82	0.51–1.31	0.408
(**B**)			
**Variable**	**HR**	**95% CI (Lower–Upper)**	***p*-Value**
ECOG-PS	2.62	1.50–4.56	0.001 *
Stage IV	2.50	0.84–7.46	0.100
Bone metastasis	0.93	0.52–1.66	0.807
Liver metastasis	1.00	0.48–2.07	0.999
Extrathoracic metastasis	1.54	0.81–2.90	0.187
>3 metastatic sites	1.38	0.72–2.63	0.336
Albumin at baseline	1.57	0.91–2.71	0.109
Age ≥ 65	0.99	0.60–1.64	0.978

CI: Confidence Interval; ECOG PS: Eastern Cooperative Oncology Group Performance Status; PFS: Progression-free survival; OS: Overall survival; * clinically significant.

**Table 7:** Immune-related adverse events (irAEs) in patients treated with Cemiplimab (n = 150).

IrAEs	Any Grade, n (%)	Grade 1–2, n (%)	Grade ≥ 3, n (%)
Any irAEs	43 (28.7)	25 (16.7)	18 (12.0)
Hepatitis	9 (6.0)	4 (2.7)	5 (3.3)
Pneumonitis	9 (6.0)	4 (2.7)	5 (3.3)
Colitis	5 (3.3)	3 (2.0)	2 (1.3)
Hypo-/hyperthyroidism	1 (0.7)	1 (0.7)	0 (0.0)
Others	19 (12.7)	13 (8.7)	6 (4.0)
**Led to discontinuation**	20 (13.3)		
**Led to death**	1 (0.7)		

## Data Availability

The original contributions presented in this study are included in the article and Supplementary Materials. Further inquiries can be directed to the corresponding author.

## Supplementary Materials

The following supporting information can be downloaded at: https://www.mdpi.com/article/10.3390/cancers17223643/s1, Table S1: Participant Hospital Cemiplimab cohort (N = 150); Table S2: Participant Hospital Pembrolizumab cohort (N = 144); Table S3: Clinical characteristics of patients in Pembrolizumab cohort; Table S4: Overall response rate according RECIST criteria by site; Table S5: PFS according to clinical characteristics in Pembrolizumab cohort; Table S6: OS according to clinical characteristics in Pembrolizumab cohort.

## Abbreviations

The following abbreviations are used in this manuscript:

AJCC	American Joint Committee on Cancer
ALK	Anaplastic Lymphoma Kinase
BMI	Body Mass Index
CI	Confidence Interval
CR	Complete Response
CTCAE	Common Terminology Criteria for Adverse Events
CTLA-4	Cytotoxic T-Lymphocyte–Associated Protein 4
dNLR	Derived Neutrophil-to-Lymphocyte Ratio
DCR	Disease Control Rate
eCRF	Electronic Case Report Form
ECLC	European Conference on Lung Cancer
ECOG	Eastern Cooperative Oncology Group
EGFR	Epidermal Growth Factor Receptor
HR	Hazard Ratio
IASLC	International Association for the Study of Lung Cancer
ICIs	Immune Checkpoint Inhibitors
IRB	Institutional Review Board
irAEs	Immune-Related Adverse Events
LIPI	Lung Immune Prognostic Index
mOS	Median Overall Survival
mPFS	Median Progression-Free Survival
NGS	Next-Generation Sequencing
NSCLC	Non–Small Cell Lung Cancer
OR	Odds Ratio
ORR	Objective Response Rate
OS	Overall Survival
PD	Progressive Disease
PD-1	Programmed Cell Death Protein 1
PD-L1	Programmed Death-Ligand 1
PFS	Progression-Free Survival
PR	Partial Response
PS	Performance Status
PS-ECOG	Performance Status by Eastern Cooperative Oncology Group
PSM	Propensity Score Matching
RECIST	Response Evaluation Criteria in Solid Tumors
ROS1	ROS Proto-Oncogene 1 (Receptor Tyrosine Kinase)
RWD	Real-World Data
SD	Stable Disease
SPSS	Statistical Package for the Social Sciences
TNM	Tumor–Node–Metastasis
TPS	Tumor Proportion Score
TRAEs	Treatment-Related Adverse Events
WCLC	World Conference on Lung Cancer
WHO	World Health Organization

## References

[1] Waldman A.D., Fritz J.M., Lenardo M.J. (2020). A guide to cancer immunotherapy: From T cell basic science to clinical practice. Nat. Rev. Immunol..

[2] Herbst R.S., Morgensztern D., Boshoff C. (2018). The biology and management of non-small cell lung cancer. Nature.

[3] Reck M., Remon J., Hellmann M.D. (2022). First-Line Immunotherapy for Non–Small-Cell Lung Cancer. J. Clin. Oncol..

[4] Hendriks L.E., Cortiula F., Mariamidze E., Martins Branco D., Pentheroudakis G., Reck M. (2025). ESMO Non-Oncogene-Addicted Non-Small Cell Lung Cancer Living Guideline v1.2. https://www.esmo.org/living-guidelines/esmo-non-oncogene-addicted-metastatic-non-small-cell-lung-cancer-living-guideline.

[5] Herbst R.S., Giaccone G., de Marinis F., Reinmuth N., Vergnenegre A., Barrios C.H., Morise M., Felip E., Andric Z., Geater S. (2020). Atezolizumab for First-Line Treatment of PD-L1-Selected Patients with NSCLC. N. Engl. J. Med..

[6] Brahmer J.R., Rodriguez-Abreu D., Robinson A.G., Hui R., Csőszi T., Fülöp A., Gottfried M., Peled N., Tafreshi A., Cuffe S. (2020). LBA51 KEYNOTE-024 5-year OS update: First-line (1L) pembrolizumab (pembro) vs platinum-based chemotherapy (chemo) in pts with metastatic NSCLC and PD-L1 tumour proportion score (TPS) ≥ 50%. Ann. Oncol..

[7] Kilickap S., Baramidze A., Sezer A., Özgüroğlu M., Gumus M., Bondarenko I., Gogishvili M., Nechaeva M., Schenker M., Cicin I. (2025). Cemiplimab Monotherapy for First-Line Treatment of Patients with Advanced NSCLC With PD-L1 Expression of 50% or Higher: Five-Year Outcomes of EMPOWER-Lung 1. J. Thorac. Oncol..

[8] Sezer A., Kilickap S., Gümüş M., Bondarenko I., Özgüroğlu M., Gogishvili M., Turk H.M., Cicin I., Bentsion D., Gladkov O. (2021). Cemiplimab monotherapy for first-line treatment of advanced non-small-cell lung cancer with PD-L1 of at least 50%: A multicentre, open-label, global, phase 3, randomised, controlled trial. Lancet.

[9] Özgüroğlu M., Kilickap S., Sezer A., Gümüş M., Bondarenko I., Gogishvili M., Nechaeva M., Schenker M., Cicin I., Ho G.F. (2023). First-line cemiplimab monotherapy and continued cemiplimab beyond progression plus chemotherapy for advanced non-small-cell lung cancer with PD-L1 50% or more (EMPOWER-Lung 1): 35-month follow-up from a multicentre, open-label, randomised, phase 3 trial. Lancet Oncol..

[10] Cortellini A., Brunetti L., Di Fazio G.R., Garbo E., Pinato D.J., Naidoo J., Katz A., Loza M., Neal J.W., Genova C. (2025). Determinants of 5-year survival in patients with advanced NSCLC with PD-L1 ≥ 50% treated with first-line pembrolizumab outside of clinical trials: Results from the Pembro-real 5Y global registry. J. Immunother. Cancer.

[11] Cortellini A., Ricciuti B., Facchinetti F., Alessi J.V.M., Venkatraman D., Dall’Olio F.G., Cravero P., Vaz V.R., Ottaviani D., Majem M. (2021). Antibiotic-exposed patients with non-small-cell lung cancer preserve efficacy outcomes following first-line chemo-immunotherapy. Ann. Oncol..

[12] Facchinetti F., Di Maio M., Perrone F., Tiseo M. (2021). First-line immunotherapy in non-small cell lung cancer patients with poor performance status: A systematic review and meta-analysis. Transl. Lung Cancer Res..

[13] Freemantle N., Xu Y., Wilson F.R., Guyot P., Chen C.I., Keeping S., Konidaris G., Chan K., Kuznik A., Atsou K. (2022). Network meta-analysis of immune-oncology monotherapy as first-line treatment for advanced non-small-cell lung cancer in patients with PD-L1 expression ≥50. Ther. Adv. Med. Oncol..

[14] Majem M., Cobo M., Isla D., Marquez-Medina D., Rodriguez-Abreu D., Casal-Rubio J., Moran-Bueno T., Bernabé-Caro R., Pérez-Parente D., Ruiz-Gracia P. (2021). PD-(L)1 Inhibitors as Monotherapy for the First-Line Treatment of Non-Small-Cell Lung Cancer Patients with High PD-L1 Expression: A Network Meta-Analysis. J. Clin. Med..

[15] Cramer-van der Welle C.M., Verschueren M.V., Tonn M., Peters B.J.M., Schramel F.M.N.H., Klungel O.H., Groen H.J.M., Van De Garde E.M.W., Kastelijn E.A., The Santeon NSCLC Study Group (2021). Real-world outcomes versus clinical trial results of immunotherapy in stage IV non-small-cell lung cancer (NSCLC) in the Netherlands. Sci. Rep..

[16] Piedra A., Martínez-Recio S., Hernández A., Morán T., Arriola E., Recuero-Borau J., Cobo M., Cordeiro P., Mosquera J., Fernández M. (2024). First-line pembrolizumab in patients with advanced non-small cell lung cancer and high PD-L1 expression: Real-world data from a Spanish multicenter study. Front. Oncol..

[17] Tambo Y., Sone T., Shibata K., Nishi K., Shirasaki H., Yoneda T., Araya T., Kase K., Nishikawa S., Kimura H. (2020). Real-World Efficacy of First-Line Pembrolizumab in Patients With Advanced or Recurrent Non-Small-Cell Lung Cancer and High PD-L1 Tumor Expression. Clin. Lung Cancer.

[18] Velcheti V., Hu X., Yang L., Pietanza M.C., Burke T. (2022). Long-Term Real-World Outcomes of First-Line Pembrolizumab Monotherapy for Metastatic Non-Small Cell Lung Cancer With ≥50% Expression of Programmed Cell Death-Ligand 1. Front. Oncol..

[19] Pelicon V., Cufer T., Knez L. (2023). Real-world outcomes of immunotherapy with or without chemotherapy in first-line treatment of advanced non-small cell lung cancer. Front. Oncol..

[20] Cortellini A., Tiseo M., Banna G.L., Cappuzzo F., Aerts J.G.J.V., Barbieri F., Giusti R., Bria E., Cortinovis D., Grossi F. (2020). Clinicopathologic correlates of first-line pembrolizumab effectiveness in patients with advanced NSCLC and a PD-L1 expression of ≥ 50. Cancer Immunol. Immunother..

[21] Cortellini A., Ricciuti B., Tiseo M., Bria E., Banna G.L., Aerts J.G., Barbieri F., Giusti R., Cortinovis D.L., Migliorino M.R. (2020). Baseline BMI and BMI variation during first line pembrolizumab in NSCLC patients with a PD-L1 expression ≥ 50%: A multicenter study with external validation. J. Immunother. Cancer.

[22] Li J.J.N., Karim K., Sung M., Le L.W., Lau S.C.M., Sacher A., Leighl N.B. (2020). Tobacco exposure and immunotherapy response in PD-L1 positive lung cancer patients. Lung Cancer.

[23] Popat S., Liu S.V., Scheuer N., Gupta A., Hsu G.G., Ramagopalan S.V., Griesinger F., Subbiah V. (2022). Association Between Smoking History and Overall Survival in Patients Receiving Pembrolizumab for First-Line Treatment of Advanced Non-Small Cell Lung Cancer. JAMA Netw. Open.

[24] de Jager V.D., Timens W., Bayle A., Botling J., Brcic L., Büttner R., Fernandes M.G.O., Havel L., Hochmair M.J., Hofman P. (2024). Developments in predictive biomarker testing and targeted therapy in advanced stage non-small cell lung cancer and their application across European countries. Lancet Reg. Health Eur..

[25] Landre T., Justeau G., Assié J.B., Chouahnia K., Davoine C., Taleb C., Chouaïd C., Duchemann B. (2022). Anti-PD-(L)1 for KRAS-mutant advanced non-small-cell lung cancers: A meta-analysis of randomized-controlled trials. Cancer Immunol. Immunother..

[26] Facchinetti F., Mazzaschi G., Barbieri F., Passiglia F., Mazzoni F., Berardi R., Proto C., Cecere F.L., Pilotto S., Scotti V. (2020). First-line pembrolizumab in advanced non-small cell lung cancer patients with poor performance status. Eur. J. Cancer.

[27] Verkerk K., Geurts B.S., Zeverijn L.J., van der Noort V., Verheul H.M.W., Haanen J.B.A.G., Van Der Veldt A.A.M., Eskens F.A.L.M., Aarts M.J.B., Van Herpen C.M.L. (2024). Cemiplimab in locally advanced or metastatic cutaneous squamous cell carcinoma: Prospective real-world data from the DRUG Access Protocol. Lancet Reg. Health Eur..

[28] Cortellini A., Friedlaender A., Banna G.L., Porzio G., Bersanelli M., Cappuzzo F., Aerts J.G.J.V., Giusti R., Bria E., Cortinovis D. (2020). Immune-related Adverse Events of Pembrolizumab in a Large Real-world Cohort of Patients With NSCLC With a PD-L1 Expression ≥ 50% and Their Relationship With Clinical Outcomes. Clin. Lung Cancer.

[29] Zhou Y., Chen H., Tang L., Feng Y., Tao Y., Huang L., Lou N., Shi Y. (2023). Association of immune-related adverse events and efficacy in advanced non-small-cell lung cancer: A systematic review and meta-analysis. Immunotherapy.

[30] Cook S., Samuel V., Meyers D.E., Stukalin I., Litt I., Sangha R., Morris D.G., Heng D.Y.C., Pabani A., Dean M. (2024). Immune-Related Adverse Events and Survival Among Patients With Metastatic NSCLC Treated With Immune Checkpoint Inhibitors. JAMA Netw. Open.

