# Supplementary material for: Real-World Data of First-Line Cemiplimab Monotherapy for Metastatic Non-Small Cell Lung Cancer (NSCLC) with PD-L1 Expression ≥ 50%: A National Spanish Multicentric Cohort (CEMI-SPA Study)
Source: Cancers (Basel). 2025 Nov 13;17(22):3643. doi: 10.3390/cancers17223643 (PMC12651785; doi:10.3390/cancers17223643)
Supplement: Supplementary file 1 [file cancers-17-03643-s001.zip › cancers-3915491-supplementary.pdf]

SUPPLEMENTARY TABLES

Supplementary Table S1. Participant Hospital Cemiplimab cohort (N = 150)

Supplementary Table S1. Participant Hospital Cemiplimab cohort (N = 150)	
**Participating Hospital**	**Number of Patients**
Hospital Universitario de Toledo	20
Hospital Universitario Donostia	19
Hospital Universitario Araba	13
Hospital Clinico Universitario San Carlos	12
Hospital Universitario de la Ribera	11
Hospital Universitario Principe de Asturias	10
Hospital Universitario del Henares	9
Hospital Universitario 12 de Octubre	7
Hospital Universitario de Torrejón	7
Hospital Universitario Infanta Sofía	7
Complexo Hospitalario Universitario de Ourense	6
Hospital del Mar - Parc de Salut Mar	5
Hospital Luis Alcanyis de Xativa	5
Hospital Universitario San Cecilio	4
Complexo Hospitalario Universitario A Coruña	3
START Madrid-FJD, Fundación Jiménez Diaz University Hospital	3
Hospital Universitario Son Espases	2
Hospital Universitario Infanta Elena	2
Hospital Universitario Ramón y Cajal	2
Hospital Universitari Sant Joan de Reus	2
Hospital Universitario de Jerez de la Frontera	1

Supplementary Table S2. Participant Hospital Pembrolizumab cohort (N = 144)

Supplementary Table S2. Participant Hospital Pembrolizumab cohort (N = 144)	
**Participating Hospital**	**Number of Patients**
Hospital Gregorio Marañón	34
Hospital Clínico San Carlos	22
Hospital 12 de Octubre	22
Hospital Ramón y Cajal	15
Hospital Fundación Alcorcón	12
Hospital Infanta Sofía	12
Hospital de Henares	11
Hospital de Torrejón	8
Hospital Severo Ochoa	5
Hospital de Móstoles	3

Supplementary Table S3. Clinical characteristics of patients in Pembrolizumab cohort

Supplementary Table S3. Clinical characteristics of patients in Pembrolizumab cohort (n = 144)	
**Variable**	**n (%)**
**Age at diagnosis (range 44–87)**	Mean 69, SD 10.23 (63.2% > 65 years)
**Sex**	
Male	108 (75.0)
Female	36 (25.0)
**ECOG PS**	
0	25 (17.4)
1	79 (54.8)
2	37 (25.7)
3	3 (2.1)
**Smoking history**	
Never smoker	5 (3.5)
Former smoker	87 (60.4)
Current smoker	52 (36.1)
**Biopsy location**	
Lung	103 (71.5)
Lymph nodes	11 (7.6)
Others	31 (21.9)
**Liver metastases**	
Yes	21 (14.6)
No	123 (85.4)
**CNS metastases**	
Yes	27 (18.8)
No	117 (81.3)
**Liver and CNS metastases**	
Yes	2 (1.4)
No	142 (98.6)
**LIPI score**	
Good	38 (26.4)
Intermediate	56 (38.9)
Poor	29 (20.1)
Missing	21 (14.6)
**dNLR > 3**	
Yes	60 (41.7)
No	84 (58.3)
**Actionable genetic alteration***	
Yes	12 (8.3)
No	132 (91.7)
**LDH > ULN**	
Yes	61 (42.4)
No	62 (43)
Missing	21 (14.6)
**Progression to 1L immunotherapy**	98 (68.1)
**Clinical benefit**	74 (51.4)
**Best response by RECIST**	

Complete response	5 (3.5)
Partial response	59 (41)
Stable disease	17 (11.8)
Progressive disease	63 (43.7)
**Vital status**	
Deceased	102 (70.8)
Disease-related death	89 (61.8)

CNS: Central Nervous System; dNLR: Derived Neutrophil-to-Lymphocyte Ratio; ECOG PS: Eastern Cooperative Oncology Group Performance Status; LIPI: Lung Immune Prognostic Index; SD: Standard Desviation; ULN: Upper Limit of Normal.

Supplementary Table S4. Overall response rate according RECIST criteria by site

Supplementary Table S4. Overall response rate (ORR) according RECIST criteria by site				
**Participating Hospital**	**Yes**	**No**	**Total**	**% Response**
Hospital Universitario de Toledo	10	7	17	58,8
Hospital Universitario Donostia	15	4	19	78,9
Hospital Universitario Araba	4	9	13	30,8
Hospital Clinico Universitario San Carlos	9	3	12	75
Hospital Universitario de la Ribera	6	5	11	54,5
Hospital Universitario Principe de Asturias	3	7	10	30
Hospital Universitario del Henares	6	3	9	66,7
Hospital Universitario 12 de Octubre	3	4	7	42,9
Hospital Universitario de Torrejón	5	2	7	71,4
Hospital Universitario Infanta Sofía	5	2	7	71,4
Complexo Hospitalario Universitario de Ourense	2	3	5	40
Hospital del Mar - Parc de Salut Mar	2	3	5	40
Hospital Luis Alcanyis de Xativa	3	2	5	60
Hospital Universitario San Cecilio	0	4	4	0
Complexo Hospitalario Universitario A Coruña	2	1	3	66,7
START Madrid-FJD, Fundación Jiménez Diaz	2	1	3	66,7
Hospital Universitario Son Espases	1	1	2	50
Hospital Universitario Infanta Elena	1	1	2	50
Hospital Universitario Ramón y Cajal	2	0	2	100
Hospital Universitari Sant Joan de Reus	2	0	2	100
Hospital Universitario de Jerez de la Frontera	1	0	1	100

Supplementary Table S5. PFS according to clinical characteristics in Pembrolizumab cohort.

Supplementary Table S5. PFS according to clinical characteristics in Pembrolizumab cohort				
**Variable**	**median PFS (95% CI, months)**	**HR**	**IC 95% (Lower - Upper)**	**p-value**
**Age**		0.74	0.49-1.11	0.14
< 65 years	3.1 (1.4–4.9)			
≥ 65 years	10.1 (5.6–14.7)			
**Sex**		1.08	0.68-1.72	0.73
Male	7.8 (3.2–12.6)			
Female	4.1 (0.3–7.9)			
**Performance status**		1.71	1.26-2.35	0.000***
0	9.6 (0.0–27.4)			
1	9.8 (2.8–16.8)			
2	3.1 (0.6–5.8)			
3	0.3 (0.0–0.2)			
**Smoking status**		0.31	0.11-0.86	0.037*
Never-smoker	1.1 (0.6–1.7)			
Former smoker	9.4 (4.1–14.8)			
Current smoker	5.3 (0.3–10.2)			
**Histology**		1.08	0.72-1.63	0.72
Adenocarcinoma	5.8 (0.0–11.7)			
Non-adenocarcinoma	6.8 (1.8–11.9)			
**PD-L1 expression**		1.33	0.87-2.03	0.18
≥50–<90%	8.5 (3.3–13.7)			
≥90%	5.8 (1.6–10.1)			
**Stage at begning pembrolizumab**		2.10	0.77-5.73	0.14
III	mPFS not reached			
IV	5.8 (1.9–9.6)			
**Prior antibiotics**		1.61	1.07-2.43	0.022*
Yes	3.1 (1.6–4.7)			
No	11.0 (5.6–16.7)			
**Liver metastases**		1.44	0.85-2.50	0.18
Yes	2.9 (2.0–3.8)			
No	8.4 (4.6–12.3)			
**CNS metastases**		1.18	0.72-1.92	0.50
Yes	3.9 (0.7–7.2)			
No	8.4 (3.8–12.9)			
**LIPI score**		0.66	0.49-0.88	0.011*
Good	15.7 (2.5–28.9)			
Intermediate	5.8 (1.8–9.9)			
Poor	1.7 (1.4–2.2)			
**dNLR ≥ 3**		1.72	1.16-2.56	0.007**
Yes	2.7 (0.5–5.0)			
No	9.8 (1.7–17.8)			
**LDH > ULN**		1.76	1.14-2.71	0.01*

Yes	2.7 (1.0–4.4)			
No	8.4 (2.2–14.8)			

CI: Confidence Interval; CNS: Central Nervous System; dNLR: Derived Neutrophil-to-Lymphocyte Ratio; ECOG PS: Eastern Cooperative Oncology Group Performance Status; LIPI: Lung Immune Prognostic Index; PFS: Progression-Free Survival; ULN: Upper Limit of Normal.

Supplementary Table S6. OS according to clinical characteristics in Pembrolizumab cohort.

**Supplementary Table S6 OS according to clinical characteristics in Pembrolizumab cohort**				
**Variable**	**Median OS (95% CI, months)**	**HR**	**IC 95% (Lower - Upper)**	**p-value**
**Age**		0.90	0.60-1.36	0.621
< 65 years	10.2 (5.8–14.6)			
≥ 65 years	12.1 (6.4–17.8)			
**Sex**		0.93	0.59-1.46	0.740
Male	10.4 (6.9–13.9)			
Female	13.8 (4.0–23.7)			
**ECOG**		2.03	1.49-2.77	0.000***
0	27.8 (10.7–45.0)			
1	14.4 (9.2–19.5)			
2	5.7 (4.0–7.4)			
3	0.1 (0.03–0.2)			
**Smoking status**		0.92	0.64-1.3	0.720
Never smoker	10.6 (0.0–21.5)			
Former smoker	12.1 (6.5–17.7)			
Current smoker	10.0 (6.7–13.4)			
**Histology**		1.45	0.98-2.16	0.064
Adenocarcinoma	13.1 (6.6–19.6)			
Non-adenocarcinoma	10.0 (7.6–12.4)			
**PD-L1 expression**		1.33	0.87-2.02	0.180
≥50–<90%	12.4 (7.1–17.7)			
≥90%	10.0 (6.7–13.4)			
**Stage at beginning pembrolizumab**		2.33	0.86-6.35	0.087
III	29.0 (2.8–51.3)			
IV	10.4 (8.0–12.8)			
**Prior antibiotics**		2.07	1.39-3.09	0.000***

Yes	6.6 (2.7–10.5)			
No	16.6 (11.4–21.8)			
**Liver metastases**		1.59	0.94-2.69	0.076
Yes	8.0 (2.3–13.8)			
No	11.7 (7.0–16.4)			
**CNS metastases**		1.01	0.60-1.70	0.981
Yes	7.6 (0.9–14.3)			
No	11.7 (8.1–15.3)			
**LIPI score**		0.67	0.50-0.88	0.010*
Good	17.4 (9.4–25.3)			
Intermediate	10.3 (7.6–13.1)			
Poor	5.0 (1.0–9.0)			
**dNLR ≥ 3**		1.60	1.08-2.36	0.017*
Yes	6.0 (3.5–8.5)			
No	12.9 (6.7–19.0)			
**LDH > ULN**		1.65	1.08-2.53	0.020*
Yes	8.2 (1.0–4.4)			
No	13.8 (7.8–19.9)			

CI: Confidence Interval; CNS: Central Nervous System; dNLR: Derived Neutrophil-to-Lymphocyte Ratio; ECOG PS: Eastern Cooperative Oncology Group Performance Status; LIPI: Lung Immune Prognostic Index; OS: Overall Survival; ULN: Upper Limit of Normal.
